# Compressive mechanical stress enhances susceptibility to interleukin-1 by increasing interleukin-1 receptor expression in 3D-cultured ATDC5 cells

**DOI:** 10.1186/s12891-021-04095-x

**Published:** 2021-03-01

**Authors:** Yuki Takeda, Yasuo Niki, Yusuke Fukuhara, Yoshitsugu Fukuda, Kazuhiko Udagawa, Masayuki Shimoda, Toshiyuki Kikuchi, Shu Kobayashi, Kengo Harato, Takeshi Miyamoto, Morio Matsumoto, Masaya Nakamura

**Affiliations:** 1 Department of Orthopaedic Surgery, School of Medicine, Keio University, 35 Shinanomachi, Shinjuku-ku, Tokyo, 160-8582 Japan; 2 Department of Pathology, School of Medicine, Keio University, Tokyo, Japan; 3 Department of Orthopaedic Surgery, Faculty of Life Sciences, Kumamoto University, Kumamoto, Japan

**Keywords:** 3D-cultured ATDC5 cells, Cyclic compressive loading, Interleukin-1, Transient receptor potential vanilloid 4

## Abstract

**Background:**

Mechanical overload applied on the articular cartilage may play an important role in the pathogenesis of osteoarthritis. However, the mechanism of chondrocyte mechanotransduction is not fully understood. The purpose of this study was to assess the effects of compressive mechanical stress on interleukin-1 receptor (IL-1R) and matrix-degrading enzyme expression by three-dimensional (3D) cultured ATDC5 cells. In addition, the implications of transient receptor potential vanilloid 4 (TRPV4) channel regulation in promoting effects of compressive mechanical loading were elucidated.

**Methods:**

ATDC5 cells were cultured in alginate beads with the growth medium containing insulin-transferrin-selenium and BMP-2 for 6 days. The cultured cell pellet was seeded in collagen scaffolds to produce 3D-cultured constructs. Cyclic compressive loading was applied on the 3D-cultured constructs at 0.5 Hz for 3 h. The mRNA expressions of a disintegrin and metalloproteinases with thrombospondin motifs 4 (ADAMTS4) and IL-1R were determined with or without compressive loading, and effects of TRPV4 agonist/antagonist on mRNA expressions were examined. Immunoreactivities of reactive oxygen species (ROS), TRPV4 and IL-1R were assessed in 3D-cultured ATDC5 cells.

**Results:**

In 3D-cultured ATDC5 cells, ROS was induced by cyclic compressive loading stress. The mRNA expression levels of ADAMTS4 and IL-1R were increased by cyclic compressive loading, which was mostly prevented by pyrollidine dithiocarbamate. Small amounts of IL-1β upregulated ADAMTS4 and IL-1R mRNA expressions only when combined with compressive loading. TRPV4 agonist suppressed ADAMTS4 and IL-1R mRNA levels induced by the compressive loading, whereas TRPV4 antagonist enhanced these levels. Immunoreactivities to TRPV4 and IL-1R significantly increased in constructs with cyclic compressive loading.

**Conclusion:**

Cyclic compressive loading induced mRNA expressions of ADAMTS4 and IL-1R through reactive oxygen species. TRPV4 regulated these mRNA expressions, but excessive compressive loading may impair TRPV4 regulation. These findings suggested that TRPV4 regulates the expression level of IL-1R and subsequent IL-1 signaling induced by cyclic compressive loading and participates in cartilage homeostasis.

**Supplementary Information:**

The online version contains supplementary material available at 10.1186/s12891-021-04095-x.

## Background

Mechanical overload applied to articular cartilage may play an important role in the pathogenesis of osteoarthritis (OA). However, the pathomechanisms of mechanical overload-induced cartilage degradation are poorly understood. The initial process of cartilage degradation has been shown to be associated with proteoglycan loss [1] and subsequent decreases in tissue hydration. As negatively charged glucosaminoglycan chains in proteoglycan draw in and maintain water within the cartilage matrix [2], proteoglycan loss decreases the interstitial osmotic pressure. Joint loading causes deformation of the cartilage layer and associated loss of interstitial water, followed by recovery of the fluid as the load is released [3–5]. The membrane stretch due to joint loading as well as osmotic pressure change reportedly elicit the activation of pumps and channels such as transient receptor potential vanilloid 4 (TRPV4), which initiates intracellular signaling cascade and changes cellular volume, designated as a regulatory volume decrease (RVD) or increase [6–11]. Although compressive stress on the cartilage results in various environmental changes, such as osmotic pressure, fluid flows and tensile stress, which lead to different biological responses [12–15], most important mechanisms underlying mechanical stress-induced cartilage degradation is overproduction of matrix-degrading enzymes, such as matrix metalloproteinase (MMPs) and a disintegrin and metalloproteinases with thrombospondin motifs 4 (ADAMTS) [16]. In this context, reactive oxygen species (ROS) reportedly acts as integral factors in intracellular signaling mechanisms to produce matrix-degrading enzymes [17].

To date, numerous studies have reported a close pathological relationship between interleukin-1β (IL-1β) and OA. Whether the chondrocyte itself possesses the capacity to produce IL-1β remains highly controversial, but high levels of caspase-1 and the active form of IL-1β have been detected in OA cartilage [18]. IL-1β has been shown to elicit transient changes in Ca^2+^ levels in chondrocytes and to affect responses to hypoosmotic stress by preventing decreases in regulatory volume [19]. Moreover, a previous study showed that expression levels of IL-1 receptor (IL-1R) doubled in OA chondrocytes as compared to those in normal chondrocytes, suggesting that susceptibility to IL-1 is increased in OA cartilage [20].

The present study used 3D cartilage constructs in a Type I collagen scaffold, since cellular phenotypes and culture conditions such as monolayer culture, three-dimensional (3D) culture, and cartilage explants influenced experimental results [21–24]. In fact, responses to mechanical loading by cells having the mature chondrocyte phenotype remain to be elucidated in the 3D environment. We developed a cyclic load bioreactor that can apply compressive deformation of the 3D constructs, mimicking the in vivo environment of human articular cartilage [25]. The bioreactor can be adopted to examine chondrocyte responses to the cyclic compressive stress. We hypothesized that IL-1 susceptibility and matrix-degrading enzyme expression would be increased by compressive mechanical stress and subsequent ROS induction, and this would be controlled by TRPV4, as a calcium channel mechanosensor that reportedly plays an important role in the regulation of cellular volume changes [26]. The purpose of this study was to assess compressive mechanical stress-induced IL-1R expression in 3D-cultured ATDC5 cells, and the effects of TRPV4 modification on IL-1R expression.

## Methods

### Cell culture

The ATDC5 mouse chondrogenic cell line was purchased from ATCC. ATDC5 cells were cultured in Dulbecco’s modified Eagle medium/Ham’s F12 (DMEM/F12) medium (Gibco BRL, Gaithersburg, MD) supplemented with 5% fetal bovine serum (FBS) (Sigma Aldrich, St. Louis, MO) and penicillin (100 μg/mL) -streptomycin (50 μg/mL) (Gibco BRL). Cell cultures were maintained at 37 °C in a humidified atmosphere of 5% CO_2_. Cells with more than 75% confluence were passaged using 0.25% trypsin (Gibco BRL).

### Cell differentiation in alginate beads culture

ATDC5 cells were suspended at a density of 2 × 10^6^ cells/ml in a 1.2% solution of sterile alginate (Kelco, Chicago, IL) in 0.15 M NaCl. The cell suspension was slowly expressed through a 22-gauge needle dropwise into a 100 mM calcium chloride solution. Beads were allowed to polymerize in this solution for 10 min before two consecutive washes with 0.15 M NaCl followed by two washes in DMEM/F12. After washing, beads containing ATDC5 cells were then placed into 12-well culture plates (10 beads/well) in 2 ml of DMEM/F12 containing 5% FBS, penicillin (100 μg/mL)-streptomycin (50 μg/mL), insulin-transferrin-selenium, recombinant human *bone morphogenetic protein* (BMP)-2 (0.25 μg/mL, R&D Systems, Minneapolis MN), and 50 μg/ml ascorbic acid. ATDC5 cells were cultured in alginate beads with the growth medium containing insulin-transferrin-selenium and BMP-2 for 6 days. Medium was changed once in three days. Medium was then removed from individual wells, and the beads were dissolved by incubation for 10–15 min at 4 °C in dissolving solution (55 mM sodium citrate, 30 mM Na_2_EDTA, 0.15 M NaCl, pH 6.8). The resulting suspension was centrifuged at 900 rpm for 10 min at 4 °C to separate the ATDC5 cells.

### Cell seeding in collagen scaffold and production of 3D-cultured constructs

The cultured cell pellet was seeded in collagen scaffolds to produce 3D-cultured constructs. In brief, cultured cells (5 × 10^5^/scaffold) were suspended in 1% collagen gel (Nitta gelatin, Osaka, Japan) on ice. The cell suspension was incorporated into collagen scaffolds (5-mm diameter and 3-mm thick, Atelocollagen Sponge Mighty; Koken, Tokyo, Japan;) using a 27-gauge needle syringe. The collagen scaffold had an interconnected pore size of 30 nm to 200 nm. The scaffolds were produced via the process of freeze-drying of 10% collagen gel and cross-linking to reinforce the mechanical property, which is similar to those of articular cartilage [25]. Cyclic compressive load of 40 kPa can apply a 10% compressive deformation of the constructs, which is stronger than physiological loading, since articular cartilage deformation under physiological loading is reportedly < 10% in human knees [27, 28].

### Cyclic compressive loading on 3D-cultured constructs

**Fig. 1:**
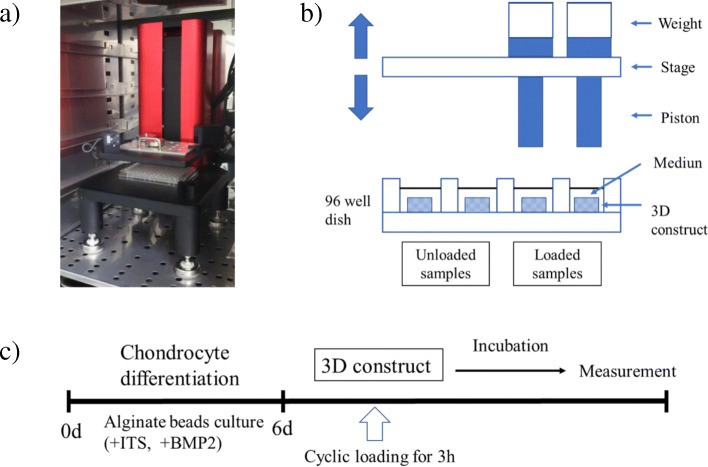
Cyclic Load Stimulator and the experimental design. **a)** Cyclic compressive loading was applied to the 3D-cultured constructs using the Cyclic Load Stimulator (Technoview, Osaka, Japan) for 3 h. **b)** Schematic representation of cyclic load stimulator. **c)** Experimental design for cyclic compressive loading on 3D-cultured constructs

Cyclic compressive loading was applied to the 3D-cultured constructs using a cyclic load stimulator (Technoview, Osaka, Japan), as previously reported [29, 30]. The loading stimulator was formed with metal plates and 96-well culture dishes where the 3D-cultured construct was incubated with DMEM/F12 and 10% FBS and maintained at a temperature of 37 °C in 5% CO_2_ (Fig. 1a). The cylindrical loading pistons connected to weights and moving stage that raises and drops the loading pistons on to the constructs can apply compressive load (Fig. 1b). In this study, cyclic compressive loading of 0 kPa, 20 kPa or 40 kPa were applied at 0.5 Hz for 3 h. The constructs at 0, 1, 3, 6 and 12 h after cyclic compressive loading of 40 kPa for 3 h were analyzed for mRNA expressions of ADAMTS4, MMP-3 and IL-1R using a real-time polymerase chain reaction (RT-PCR). The mRNA expressions of ADAMTS4, MMP-3, IL-6, IL-1β and IL-1R were quantitatively measured at 6 h after cyclic compressive loading of 0 kPa, 20 kPa and 40 kPa for 3 h (Fig. 1c).

### Effects of antioxidant, IL-1β and TRPV4 agonist/antagonist on IL-1R and ADAMTS4 mRNA expressions by 3D-cultured constructs under cyclic compressive loading

Mechanical stress induces ROS accumulation in chondrocytes in vivo [31]. ROS signaling is a risk factor for osteoarthritis [32]. To assess effects of the 3D-cultured constructs with or without mechanical loading, we evaluated ROS accumulation by staining with 8-hydroxy-2′ -deoxyguanosine (8OHdG). In nuclear and mitochondrial DNA, 8-OHdG is one of the predominant forms of free radical-induced oxidative lesions, and has therefore been widely used as a biomarker for oxidative stress and carcinogenesis [33, 34]. 3D-cultured constructs without loading were served as a control. Just before cyclic compressive loading was applied for 3 h, 50 μM of pyrollidine dithiocarbamate (PDTC) (Sigma Aldrich), an antioxidant, was added to the 3D-cultured constructs. PDTC, an inhibitor of NF-κB, affects induction of G1 phase cell cycle arrest, and prevent induction of ROS [35, 36]. The mRNA expression of ADAMTS4 and IL-1R were measured 6 h after the cyclic loading. IL-1β was purchased from R&D Systems and TRPV4 agonist (GSK1016790A) and antagonist (HC-067047) were purchased from WAKO (Osaka, Japan). IL-1β (1 pg/ml, 10 ng/ml), GSK1016790A (1 mM), or HC-067047 (1 mM) were added to the 3D-cultured construct with or without cyclic compressive loading. The mRNA expression of ADAMTS4 and IL-1R were measured 6 h after the cyclic loading.

#### Real-time PCR analysis

Total RNAs were isolated from each sample by RNAiso plus (Takara Bio, Kyoto, Japan), and cDNAs were synthesized from total RNAs using a Prime Script RT H reagent kit (Takara Bio). Real-time PCR was performed using SYBR Premix ExTaq IIH (Takara Bio) with a DICE thermal cyclerH (Takara Bio), according to the instructions from the manufacturer. Results were normalized to glyceraldhyde-3-phosphate dehydrogenase (GAPDH) as the fold change compared with samples.

The primers used were as follows: GAPDH (forward) CTT TGT CAA GCT CAT TTC CTG G, (reverse) TCT TGC TCA GTG TCC TTG C; MMP-3 (forward) GAT GAA CGA TGG ACA GAG GAT G, (reverse) AAA CGG GAC AAG TCT GTG G; ADAMTS4 (forward) CTG GGT ATG GCT GAT GTG G, (reverse) AGT GCA TGG CTT GGA GTT ATC; IL-1R1 (forward) AGT TTT CGT TTT AAC AGC CAG TG, (reverse) GAG ACA AAT GAG CCC CAG TAG; IL-6 (forward) CCA CTC ACC TCT TCA GAA CG, (reverse) CAT CTT TGG AAG GTT CAG GTT G; IL-1β (forward) ACG GAC CCC AAA AGA TGA AG, (reverse) TTC TCC ACA GCC ACA ATG AG.

### Histological assessment and immunohistochemistry

For histological examination, the 3D-cultured constructs with or without loading were fixed in 10% formalin solution and dehydrated in paraffin. Six-micrometer sections were cut using a microtome and mounted on glass slides. The sections were stained with toluidine blue. The constructs were analyzed for the presence and localization of chondroitin 4-sulfate, keratan sulfate, ROS, TRPV4 and IL-1R using immunohistochemistry. Deparaffinized and hydrated sections were incubated in methanol containing 0.3% H_2_O_2_ for 30 min. Sections were then incubated with anti-chondroitin 4-sulfate (2B6) (Cosmo Bio Co., LTD), anti-keratan sulfate (5D4) (Cosmo Bio Co., LTD), anti- 8-OHdG (1:100 dilution; JaICA, Shizuoka, Japan), anti-TRPV4 antibody (ab94868, 1:500 dilution; Abcam, Cambridge, MA) or anti-IL-1R antibody (ab106278, 1:500 dilution; Abcam). After washing with PBS, sections were incubated with the secondary antibody biotinylated anti-mouse IgG included in the ABC staining system (Vectastain Elite ABC kit; Vector Laboratories, Burlingame, CA). For sections of 3D-cultured constructs, biotinylated secondary antibody (Anti mouse IgG, Vector Laboratories, Inc.USA) and DyLight 488 streptavidin (Vector Laboratories, Inc. USA) were then used to visualize the sections. After washing, sections were incubated with diaminobenzidine (DAB substrate) and counterstained with hematoxylin. Slides were viewed under microscopy (Axio Image A1; ZEISS, Oberkochen, Germany).

#### Statistical analysis

Data are presented as mean ± standard deviation (SD). Analysis of variance (one or two factor ANOVA) was used to test for differences among more than two groups. Post hoc test was performed by a Bonferroni method. The comparisons of two groups were performed by unpaired two-tailed Student’s t-test. Each experiment was performed more than three times. *P* < 0.05 was considered to indicate statistical significance.

## Results

### Chondrogenic ATDC5 cells seeded in the type-I collagen scaffold

**Fig. 2:**
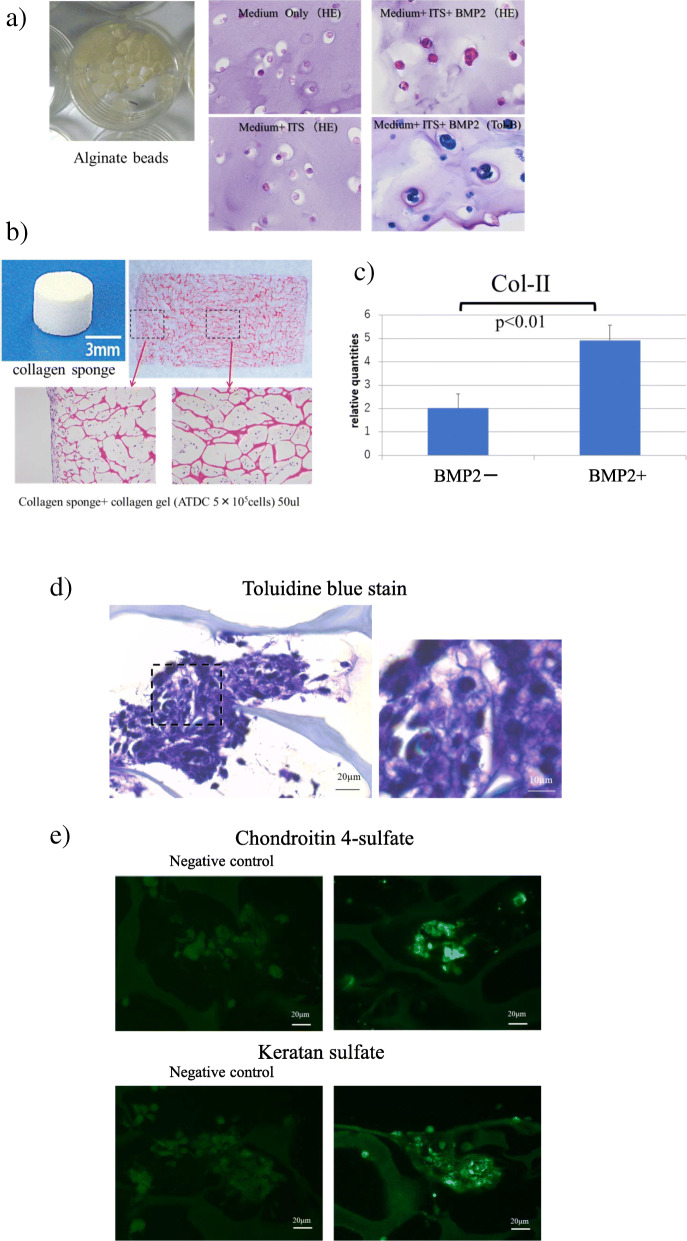
Alginate beads culture and production of 3D-cultured constructs. **a)** Alginate bead culture. ATDC5 cells were cultured in alginate beads with growth medium containing insulin-transferrin-selenium and BMP-2 for 6 days. Toluidine blue staining showed increased cartilage matrix synthesis, especially as proteoglycan in the alginate beads. **b)** After dissolving with sodium citrate and Na_2_EDTA, cells were seeded within collagen gels and incorporated into type-I collagen scaffold (Koken, Osaka, Japan) using a 27-gauge needle syringe. ATDC5 cells were distributed within the scaffold. **c)** Collagen type-II mRNA expression in the scaffold was significantly increased by the addition of recombinant BMP-2. **d)** In toluidine blue staining of 3D-cultured constructs, extracellular matrix was stained violet. **e)** In immunostaining, positive cells for the cartilage matrix components chondroitin 4-sulfate and keratan sulfate were present

ATDC5 cells were cultured in alginate beads with the growth medium containing insulin-transferrin-selenium and BMP-2 for 6 days. Toluidine blue staining revealed increased proteoglycan synthesis (Fig. 2a). After dissolving with sodium citrate and Na_2_EDTA, cells were successfully incorporated into type-I collagen scaffold (Fig. 2b). The collagen type-II mRNA expression in the scaffold was significantly increased with the addition of recombinant BMP-2 (Fig. 2c). In toluidine blue staining of 3D-cultured constructs, extracellular matrix was stained violet (Fig. 2d). In immunostaining, positive cells for the cartilage matrix components chondroitin 4-sulfate and keratan sulfate were present in 3D-cultured constructs (Fig. 2e).

### Effects of cyclic compressive loading on 3D-cultured constructs

**Fig. 3:**
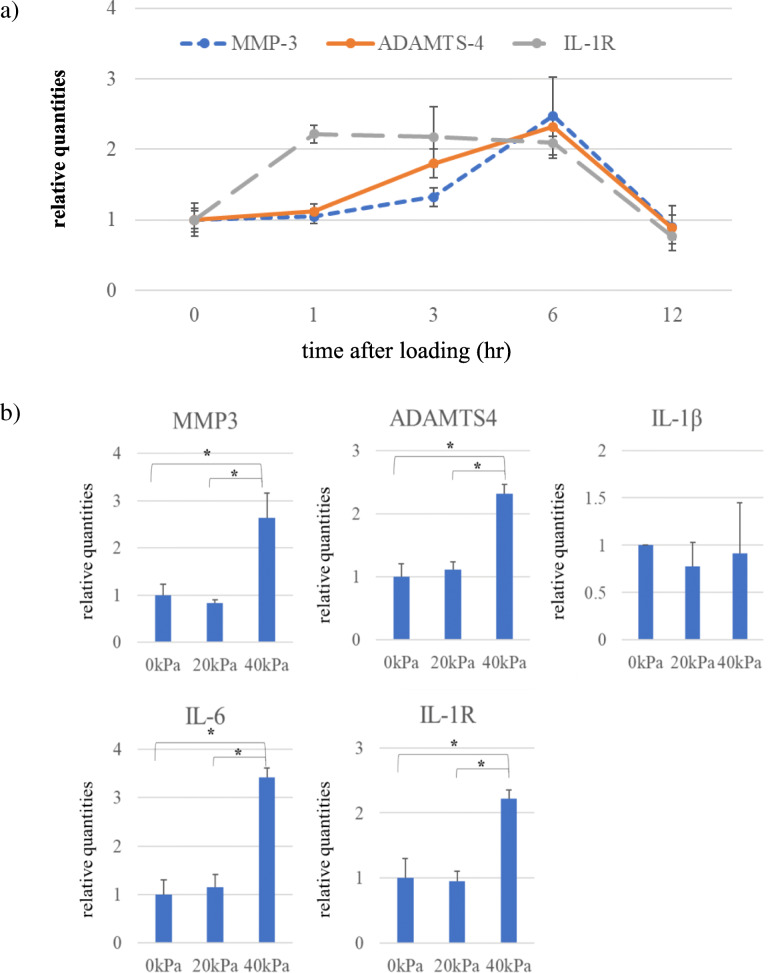
Effects of cyclic compressive loading on 3D-cultured constructs. **a)** Temporal changes in mRNA expression levels of MMP-3, ADAMTS4 and IL-1R were analyzed for 12 h (*n* = 6 each). All mRNA expression levels were upregulated at 6 h after compressive loading for 3 h. **b)** The mRNA expression levels of ADAMTS4, MMP3, IL-6, and IL-1R were all increased by 40 kPa compressive loading at 6 h, whereas these mRNA expressions were not stimulated by 20 kPa loading. IL-1β expression level was unchanged after compressive loading. Values are given as mean ± SD (*n* = 6 each). *P < 0.05

**Fig. 4:**
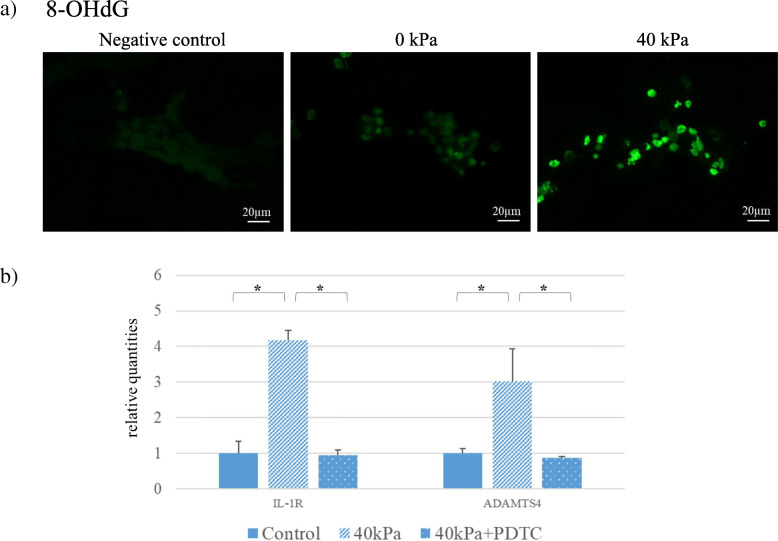
ROS accumulation in 3D-cultured constructs following cyclic compressive loading and effects of antioxidant. 3D-cultured constructs with or without cyclic loading were fixed. Cells were then stained with mouse-anti-8OHdG antibody and observed under a microscope. Bar, 20 μm. **b)** PDTC, an antioxidant, markedly prevented both ADAMTS4 and IL-1R mRNA expressions induced by compressive mechanical stress. Values are given as mean ± SD (*n* = 6 each). *P < 0.05

Time courses of expression levels of MMP-3, ADAMTS4 and IL-1R mRNAs after cyclic compressive loading of 40 kPa for 3 h were examined (Fig. 3A). MMP-3 and ADAMTS4 mRNAs were gradually increased over the first 6 h and thereafter decreased to the control level by 12 h. The IL-1R mRNA expression level was increased by 1 h after loading, maintained until 6 h, and then decreased to the control level by 12 h. The mRNA expression levels of ADAMTS4, MMP3, IL-6, and IL-1R were all increased by 40 kPa of compressive loading at 6 h, whereas such increase mRNA expressions were not observed by 20 kPa of loading (Fig. 3b). IL-1β mRNA expression was unchanged, regardless of the amount of compressive loading. The 3D-cultured constructs with cyclic compressive loading exhibited ROS accumulation, whereas those without cyclic compressive loading did not (Fig. 4a). PDTC, an antioxidant, markedly prevented mechanically induced mRNA expressions of ADAMTS4 and IL-1R (Fig. 4b).

### Increased IL-1 susceptibility of 3D-cultured constructs by cyclic compressive loading

**Fig. 5:**
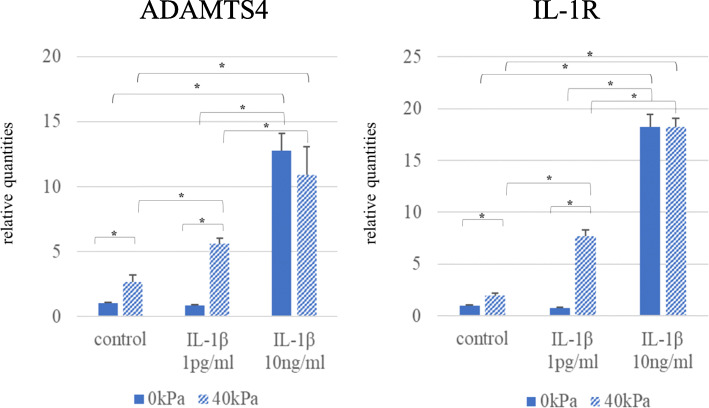
Effects of a slight amount of IL-1β with cyclic compressive loading. The mRNA levels of ADAMTS4 and IL-1R were substantially increased by the cyclic compressive stress. Increased expressions of both mRNAs were observed in the 3D-cultured constructs treated with a sufficient amount of IL-1β (10 ng/ml) as a positive control. Compressive stress plus 1 pg/ml IL-1β upregulated the levels of ADAMTS4 and IL-1R expression, but 1 pg/ml IL-1β alone failed to do so. Values are given as mean ± SD (*n* = 6 each). *P < 0.05

Increased expressions of ADAMTS4 and IL-1R mRNA were observed when 3D-cultured constructs were treated with a sufficient amount of IL-1β (10 ng/ml) in the absence of compressive loading (Fig. 5). Although a slight amount of IL-1β (1 pg/ml) alone failed to upregulate expression levels of ADAMTS4 and IL-1R mRNA, a slight amount of IL-1β (1 pg/ml) in combination with 40-kPa compressive loading dramatically increased both ADAMTS4 and IL-1R mRNA expressions by 5- and 8-fold, respectively.

### Effects of TRPV4 channel modification on ADAMTS4 and IL-1R mRNA expression levels in 3D-cultured constructs

**Fig. 6:**
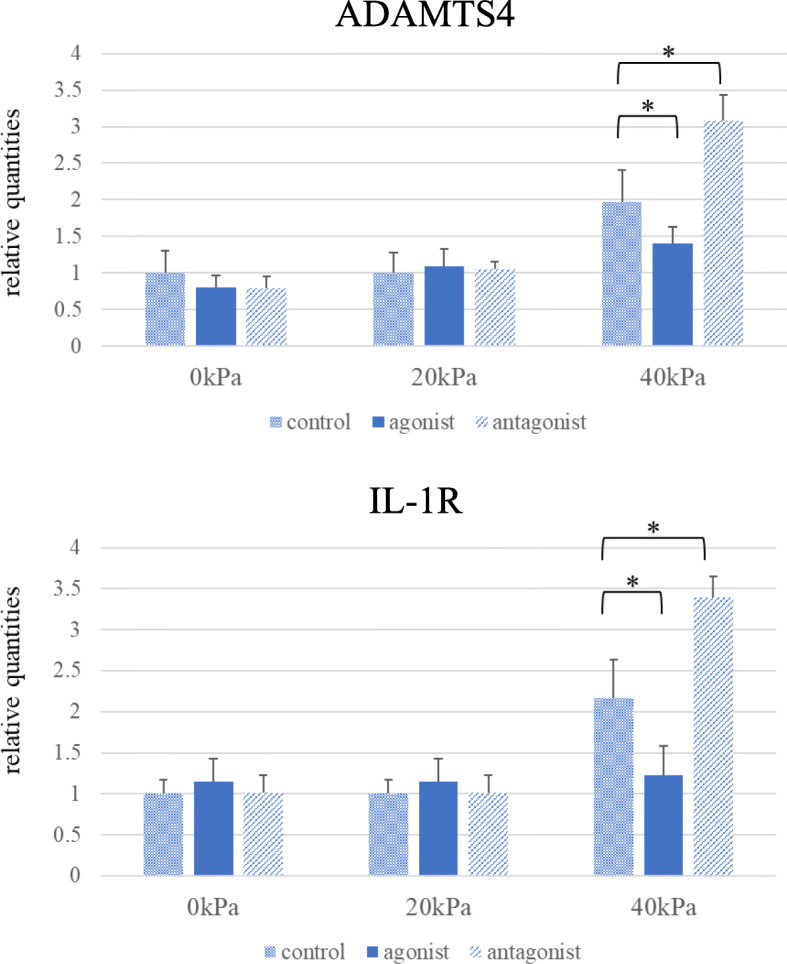
TRPV4 channel regulation in 3D-cultured constructs. TRPV4 channel regulation modulated mRNA levels of ADAMTS4 and IL-1R in 3D-cultured constructs under compressive stress. TRPV4 agonist suppressed upregulation of ADAMTS4 and IL-1R mRNAs by 40 kPa of cyclic compressive stress, whereas TRPV4 antagonist rather accelerated these mRNA expressions. Such change in both mRNA expressions were not induced by 20 kPa of cyclic compressive loading. Values are given as mean ± SD (*n* = 6 each). *P < 0.05

Neither TRPV4 agonist nor antagonist affected expression levels of ADAMTS4 and IL-1R mRNA in the absence of cyclic compressive loading. However, TRPV4 agonist suppressed upregulation of ADAMTS4 and IL-1R mRNA levels induced by 40 kPa of cyclic compressive loading. TRPV4 antagonist conversely accelerated both ADAMTS4 and IL-1R mRNA expressions. Increased expressions of the both mRNAs were not observed by 20 kPa of cyclic compressive loading (Fig. 6).

### Immunoreactivities of TRPV4 and IL-1R increase with cyclic compressive loading

**Fig. 7:**
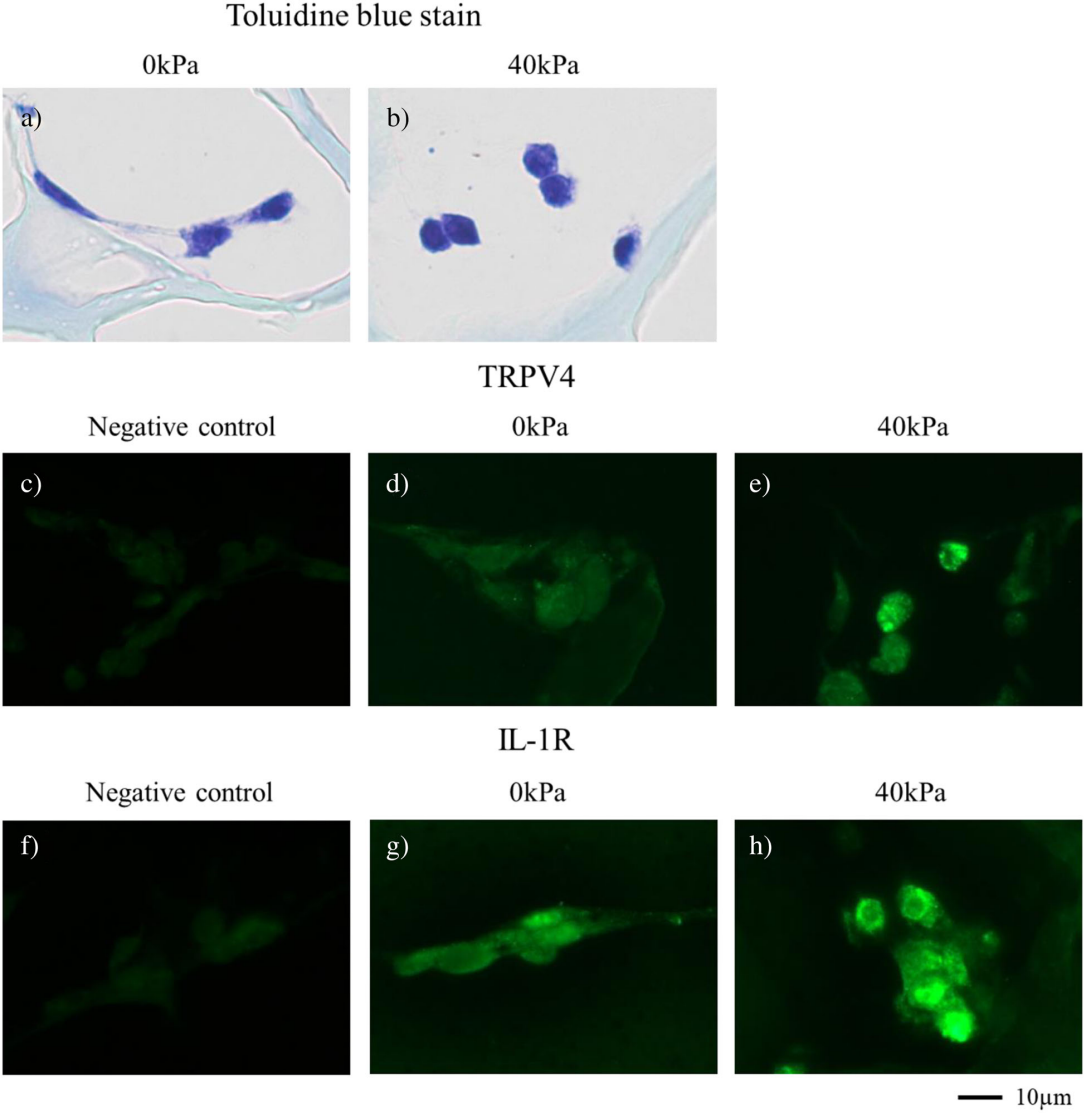
Immunohistochemistry of TRPV4 and IL-1R. a, b) In 3D-cultured constructs in type-I collagen scaffold stained with toluidine blue, cells with cyclic compressive loading showed a round morphology as compared with cells without cyclic compressive loading. c-h) Immunofluorescent staining of TRPV4 (d and e) and IL-1R (g and h) revealed significant increases in TRPV4 (e) and IL-1R (h) immunoreactivities after cyclic compressive load was applied on the 3D-cultured constructs

The 3D-cultured constructs in the type-I collagen scaffold stained with toluidine blue exhibited a round morphology after applying cyclic compressive loading, as compared to those without cyclic compressive loading serving as controls (Fig. 7a, b). The Cells extended their cell protrusions at 0 kPa and the cell protrusions disappeared at 40 kPa in 3D-cultured constructs. The cells tended to exhibit a round shape, which is a morphology resistant to compressive mechanical stress (Supple Fig). Immunoreactivities to TRPV4 (Fig. 7d, e) and IL-1R (Fig. 7g, h) significantly increased in constructs with cyclic compressive loading (Fig. 7e, h), but not in constructs without cyclic compressive loading (Fig. 7d, g).

## Discussion

Development of anti-OA therapy has been focused on the mechanisms of interaction between physical factors (i.e., mechanical or osmotic) and biochemical factors, including inflammatory cytokines and cartilage matrix-degrading enzymes, such as IL-1, ADAMTS, and MMPs. Hyper-osmotic stress has been implicated as a potential surrogate for static compressive loading in cartilage [37], and the osmotic environment plays an important role in controlling the synthesis and breakdown of components of the extracellular matrix [38]. Previous studies have suggested that the effects of static compression on cartilage was attributed to IL-1 action [39] and osmotic pressure acted synergistically with IL-1 to stimulate cyclo-oxygenase-2 and prostaglandin E2 productions in cartilage [40].

In the present study, cyclic compressive loading induced expression of IL-1R and ADAMTS4 genes through ROS induction by 3D-cultured ATDC5 cells, and that process was suppressed by TRPV4 agonist and enhanced by TRPV4 antagonist. TRPV4 has been known as a calcium channel receptor, transporting Ca^2+^ intracellularly and regulating cell volume. In steady state, physiological mechanical loading may activate TRPV4 and stimulate the chondrocyte to elicit a Ca^2+^ influx, causing regular RVD response [41]. Compression loading lead to mobilization of chondrocyte intracellular Ca^2+^ and to changes of cartilage synthesis [42, 43]. Lewis et al. showed that loss of chondrocyte volume regulation is initially a consequence of cartilage degeneration [44]. In our study, 20 kPa of loading condition, in which TRPV4 may promote normal RVD response, did not stimulate production of IL-1R or ADAMTS4, whereas a 40 kPa loading increased IL-1R production and this may result in compromising cell volume regulation by TRPV4. In fact, TRPV4 is highly expressed in articular chondrocytes, and loss of TRPV4 function is reportedly associated with joint arthropathy and osteoarthritis [45, 46]. The biological activity of IL-1 reportedly stabilizes intracellular F-actin [47] and increases cellular stiffness and viscosity [48]. TRPV4-induced Ca2+ influx counteracts the F-actin stabilization evoked by IL-1 receptor signaling [49]. Mechanical overload-induced IL-1R thus carries a risk of obstructing the normal RVD response by F-actin-induced increments in cellular stiffness, as increased cellular stiffness was accompanied by impairment of channel receptors at the cell membrane [50, 51]. Collectively, increased IL-1 susceptibility caused by mechanical overload may further impair cell volume regulation of TRPV4 and increase the risk of OA development, compatible with our results for 3D-cultured ATDC5 cells in the type-I collagen scaffold.

Mechanical signal transduction appears to be triggered by a diverse array of biophysical conditions, such as cell deformation [52], alternation of hydrostatic pressure [53], fluid flow [54], and changes in extra- or intracellular osmotic pressure [55]. Numerous studies dealing with mechanotransduction in cartilage have introduced cyclic tensile stress in the monolayer culture and hydrostatic pressure in 3D culture [56, 57]. The former experimental condition of tensile mechanical stress applied on a monolayer culture potentially causes dedifferentiation of chondrocytes. The present study utilized cyclic compressive stress of 40 kPa at 0.5 Hz, aiming at 10% deformity using the specific bioreactor, which might physiologically mimic the overloaded condition of the human knee joint. Moreover, ATDC5 cells gained the mature chondrocyte phenotype using quick alginate bead embedding followed by culture in collagen gel and type-I collagen scaffold. Such a culture environment may provide optimal experimental conditions for assessing the effects of compressive mechanical loading on mature chondrocytes.

Although whether chondrocytes possess the ability to produce IL-1β in OA cartilage remains disputable, several reports in the literature have provided positive evidence for slight amounts of IL-1β (< 1 pg/ml) in the synovial fluid of OA joints [58–60]. Indeed, subtle amount of IL-1β is assumed to be produced by surrounding synoviocytes and to gain access to and bind IL-1R on the surface of chondrocytes, and to exert biological activities as a proinflammatory cytokine. In our experiments, 1 pg/ml of IL-1β stimulated production of IL-1R and ADAMTS4 in combination with 40 kPa of cyclic compressive loading, because excessive mechanical stress substantially induced IL-1R in 3D-cultured ATDC5 cells. Another in vivo study in mice supported a critical role of IL-1R in the development of arthritis. Kagari et al. showed that arthritis induced by anti-type II collagen antibody was unable to be reproduced in IL-1R-KO mice [61]. In addition, a recent study demonstrated that IL-1R gene polymorphisms were associated with susceptibility to knee OA in the Chinese population [62]. Taken together with this evidence, IL-1R expression may make the cells more susceptible to IL-1β signaling, potentially leading to increased cartilage degeneration and OA. Finally, there are several limitations to this study. First, ATDC5 cells rather than actual chondrocytes were differentiated and embedded in a Type I collagen scaffold to create a cartilage tissue. However, responsiveness to the mechanical stress might be different from the actual articular cartilage. Second, the effects of fluid flow by piston moving of the load stimulator was not taken into account in the unloading samples. Third, protein production levels of IL-1R, ADAMTS4 and other molecules were not evaluated in this study. We assumed that compression stress causes the cell volume change, and TRPV4 may act as a cell volume regulator. If so, Ca^2+^ influx would occur, however, we have not directly analyzed Ca^2+^ influx and cell volume change. Last, the direct relationship between ROS production and TRPV4 function in response to compressive mechanical stress remains unclear. It is undoubtedly a subject of our further studies.

In summary, cyclic compressive stress is capable of stimulating expression of IL-1R and matrix-degrading enzymes such as ADAMTS4 potentially through reactive oxygen species in 3D-cultured ATDC5 cells. When the degree of compressive stress is physiological, cell volume regulation by TRPV4 may function and abrogate further downstream events, but excessive compressive stress may impair TRPV4 function and subsequently increase production of IL-1R and ADAMTS4. In the articular cartilage at the initial OA stage, proteoglycan loss begins and interstitial osmotic pressure starts decreasing. In this context, even physiological mechanical stress carries a risk of causing dysfunction of cell volume control and further progression of OA. From the perspective of mechanical loading, calcium channel receptors such as TRPV4 is a key mechanosensor and osmosensor and plays an important role not only in the regulation of cell deformation and osmotic pressure, but also in suppression of the subsequent catabolic reaction of chondrocytes. Understanding chondrocyte responses to excessive mechanical stress may provide new insights into the pathogenesis of and future therapeutic strategies for OA.

## Conclusions

Cyclic compressive loading induced mRNA expressions of ADAMTS4 and IL-1R through ROS in 3D-cultured ATDC5 cells, which was regulated by TRPV4. Excessive compressive loading may impair TRPV4 regulation. These findings suggested that TRPV4 regulates the expression level of IL-1R and subsequent IL-1 signaling induced by cyclic compressive loading and participates in cartilage homeostasis.

## Supplementary Information

**Additional file 1.**

## Data Availability

The analyzed data sets generated during the study are available from the corresponding author on reasonable request.

## Abbreviations

OA: Osteoarthritis
TRPV4: Transient receptor potential vanilloid 4
RVD: Regulatory volume decrease
MMPs: Matrix metalloproteinase
ADAMTS: A disintegrin and metalloproteinases with thrombospondin motifs
3D: Three-dimensional
ROS: Reactive oxygen species
IL-1β: Interleukin-1β
IL-1R: IL-1 receptor
BMP: Bone morphogenetic protein
RT-PCR: Real-time polymerase chain reaction
PDTC: Pyrollidine dithiocarbamate
GAPDH: Glyceraldhyde-3-phosphate dehydrogenase
DAB: Diaminobenzidine

## References

[1] Sauerland K, Raiss RX, Steinmeyer J (2003). Proteoglycan metabolism and viability of articular cartilage explants as modulated by the frequency of intermittent loading. Osteoarthr Cartil.

[2] Mastbergen SC, Bijlsma JW, Lafeber FP (2008). Synthesis and release of human cartilage matrix proteoglycans are differently regulated by nitric oxide and prostaglandin-E2. Ann Rheum Dis.

[3] Mow VC, Holmes MH, Lai WM (1984). Fluid transport and mechanical properties of articular cartilage: a review. J Biomech.

[4] Soltz MA, Ateshian GA (1998). Experimental verification and theoretical prediction of cartilage interstitial fluid pressurization at an impermeable contact interface in confined compression. J Biomech.

[5] Eckstein F, Lemberger B, Stammberger T, Englmeier KH, Reiser M (2000). Patellar cartilage deformation in vivo after static versus dynamic loading. J Biomech.

[6] Guizouarn H, Motais R, Garcia Romeu F, Borgese F (2000). Cell volume regulation: the role of taurine loss in maintaining membrane potential and cell pH. J Physiol.

[7] Hoffmann EK, Dunham PB (1995). Membrane mechanisms and intracellular signaling in cell volume regulation. Int Rev Cytol.

[8] Lange K (2000). Regulation of cell volume via microvillar ion channels. J Cell Physiol.

[9] O'Neill C (1999). Physiological significance of volume-regulatory transporters. Am J Phys.

[10] Perlman DF, Goldstein L (1999). Organic osmolyte channels in cell volume regulation in vertebrates. J Exp Zool.

[11] Waldegger S, Steuer S, Risler T, Heidland A, Capasso G, Massry S (1998). Mechanisms and clinical significance of cell volume regulation. Nephrol Dial Transplant.

[12] Chowdhury TT, Bader DL, Lee DA (2003). Dynamic compression counteracts IL-1β-induced release of nitric oxide and PGE2 by superficial zone chondrocytes cultured in agarose constructs. Osteoarthr Cartil.

[13] Guilak F, Ratcliffe A, Mow VC (1995). Chondrocyte deformation and local tissue strain in articular cartilage: a confocal microscopy study. J Orth Res.

[14] Kim Y-J, Bonassar LJ, Grodzinsky AJ (1995). The role of cartilage streaming potential, fluid flow and pressure in the stimulation of chondrocyte biosynthesis during dynamic compression. J Biomech.

[15] Chen C, Wei X, Lv Z, Sun X, Wang S, Zhang Y (2014). Primary cilia disassembly down-regulates mechanosensitive hedgehog signalling: a feedback mechanism controlling ADAMTS-5 expression in chondrocytes. Osteoarthr Cartil.

[16] Tetsunaga T, Nishida K, Furumatsu T, Naruse K, Hirohata S, Yoshida A (2011). Regulation of mechanical stress-induced MMP-13 and ADAMTS-5 expression by RUNX-2 transcriptional factor in SW1353 chondrocyte-like cells. Osteoarthr Cartil.

[17] Henrotin YE, Bruckner P, Pujol JP (2003). The role of reactive oxygen species in homeostasis and degradation of cartilage. Osteoarthr Cartil.

[18] Attur MG, Dave MN, Leung MY, Cipolletta C, Meseck M, Woo SL (2002). Functional genomic analysis of type II IL-1beta decoy receptor: potential for gene therapy in human arthritis and inflammation. J Immunol.

[19] Haider MA, Schugart RC, Setton LA, Guilak F (2006). A mechano-chemical model for the passive swelling response of an isolated chondron under osmotic loading. Biomech Model Mechanobiol.

[20] Martel-Pelletier J, Mccollum R, Dibattista J, Faure MP, Chin JA, Fournier S (1992). The interleukin-1 receptor in normal and osteoarthritic human articular chondrocytes. Arthritis Rheum.

[21] Torzilli PA, Bhargava M, Park S, Chen CT (2010). Mechanical load inhibits IL-1 induced matrix degradation in articular cartilage. Osteoarthr Cartil.

[22] Tomiyama T, Fukuda K, Yamazaki K, Hashimoto K, Ueda H, Mori S (2007). Cyclic compression loaded on cartilage explants enhances the production of reactive oxygen species. J Rheumatol.

[23] Bougault C, Gosset M, Houard X, Salvat C, Godmann L, Pap T (2012). Stress-induced cartilage degeneration dose not depend on the NLRP3 inflammasome in human osteoarthritis and mouse models. Arthritis Rheum.

[24] Xing Y, Gu Y, Gomes RR, You J (2011). P2Y(2) receptors and GRK2 are involved in oscillatory fluid flow induced ERK1/2 responses in chondrocytes. J Orthop Res.

[25] Shimomura K, Kanamoto T, Kita K, Akamine Y, Nakamura N, Mae T (2014). Cyclic compressive loading on 3D tissue of human synovial fibroblasts upregulates prostaglandin E2 via COX-2 production without IL-1β and TNF-α. Bone Joint Res.

[26] Pritchard S, Votta BJ, Kumar S, Guilak F (2008). Interleukin-1 inhibits osmotically induced calcium signaling and volume regulation in articular chondrocytes. Osteoarthr Cartil.

[27] Sutter EG, Widmyer MR, Utturkar GM, Spritzer CE, Garrett WE, DeFrate LE (2015). In vivo measurement of localized tibiofemoral cartilage strains in response to dynamic activity. Am J Sports Med.

[28] Paranjape CS, Cutcliffe HC, Grambow SC, Utturkar GM, Collins AT, Garrett WE (2019). A new stress test for knee joint cartilage. Sci Rep.

[29] Muroi Y, Kakudo K, Nakata (2007). Effects of compressive loading on human synovium-derived cell. J Dent Res.

[30] Akamine Y, Kakudo K, Kondo M, Ota K, Muroi Y, Yoshikawa H (2012). Prolonged matrix metalloproteinase-3 high expression after cyclic compressive load on human synovial cells in three-dimensional cultured tissue. Int J Oral Maxillofac Surg.

[31] Kaneko Y, Tanigawa N, Sato Y, Kobayashi T, Nakamura S, Ito E (2019). Oral administration of N-acetyl cysteine prevents osteoarthritis development and progression in a rat model. Sci Rep.

[32] Lepetsos P, Papavassiliou AG (2016). ROS/oxidative stress signaling in osteoarthritis. Biochim Biophys Acta.

[33] Valavanidis A, Vlachogianni T, Fiotakis C (2009). 8-Hydroxy-20-deoxyguanosine (8-OHdG): a critical biomarker of oxidative stress and carcinogenesis. J Environ Sci Heal Part C Environ Carcinog Ecotoxicol Rev.

[34] Pilger A, Rudiger HW (2006). 8-Hydroxy-2′-deoxyguanosine as a marker of oxidative DNA damage related to occupational and environmental exposures. Int Arch Occup Environ Health.

[35] Moon SK, Jung SY, Choi YH, Lee YC, Patterson C, Kim CH (2004). PDTC, metal chelating compound, induces G1 phase cell cycle arrest in vascular smooth muscle cells through inducing p21Cip1 expression: involvement of p38 mitogen activated protein kinase. J Cell Physiol.

[36] Jin XT, Song L, Liu X, Chen M, Li Z, Cheng L (2014). Protective efficacy of vitamins C and E on p,p'-DDT-induced cytotoxicity via the ROS-mediated mitochondrial pathway and NF-κB/FasL pathway. PLoS One.

[37] Schneiderman R, Keret D, Maroudas A (1986). Effects of mechanical and osmotic pressure on the rate of glycosaminoglycan synthesis in the human adult femoral head cartilage: an in vitro study. J Orthop Res.

[38] Urban JP, Hall AC, Gehl KA (1993). Regulation of matrix synthesis rates by the ionic and osmotic environment of articular chondrocytes. J Cell Physiol.

[39] Murata M, Bonassar LJ, Wright M, Mankin HJ, Towle CA (2003). A role for the interleukin-1 receptor in the pathway linking static mechanical compression to decreased proteoglycan synthesis in surface articular cartilage. Arch Biochem Biophys.

[40] Xing R, Wang P, Zhao L, Xu B, Zhang N, Li X (2017). Mechanism of TRPA1 and TRPV4 participating in mechanical Hyperalgesia of rat experimental knee osteoarthritis. Arch Rheumatol.

[41] Phan MN, Leddy HA, Votta BJ, Kumar S, Levy DS, Lipshutz DB (2009). Functional characterization of TRPV4 as an osmotically sensitive Ion Channel in articular chondrocytes. Arthritis Rheum.

[42] Leipzig ND, Athanasiou KA (2008). Static compression of single chondrocytes catabolically modifies single-cell gene expression. Biophys J.

[43] Mouw JK, Connelly JT, Wilson CG, Michael KE, Levenston ME (2007). Dynamic compression regulates the expression and synthesis of chondrocyte-specific matrix molecules in bone marrow stromal cells. Stem Cells.

[44] Lewis R, Feetham CH, Barrett-Jolley R (2011). Cell volume regulation in chondrocytes. Cell Physiol Biochem.

[45] Clark AL, Votta BJ, Kumar S, Liedtke W, Guilak F (2010). Chondroprotective role of the osmotically-sensitive ion channel TRPV4: age- and sex-dependent progression of osteoarthritis in Trpv4 deficient mice. Arthritis Rheum.

[46] O’Conor CJ, Leddy HA, Benefield HC, Liedtke WB, Guilak F (2014). TRPV4-mediated mechanotransduction regulates the metabolic response of chondrocytes to dynamic loading. Proc Natl Acad Sci U S A.

[47] Pritchard S, Guilak F (2006). Effects of Interleukin-1 on calcium signaling and the increase of filamentous actin in isolated and in situ articular chondrocytes. Arthritis Rheum.

[48] Pritchard S, Erickson GR, Guilak F (2002). Hyperosmotically induced volume change and calcium signaling in intervertebral disk cells: the role of the actin cytoskeleton. Biophys J.

[49] Erickson GR, Alexopoulos LG, Guilak F (2001). Hyper-osmotic stress induces volume change and calcium transients in chondrocytes by transmembrane, phospholipid, and G-protein pathways. J Biomech.

[50] Henson JH (1999). Relationships between the actin cytoskeleton and cell volume regulation. Micros Res Tech.

[51] Moustakas A, Theodoropoulos PA, Gravanis A, Haussinger D, Stournaras C (1998). The cytoskeleton in cell volume regulation. Contrib Nephrol.

[52] Madden RM, Han SK, Herzog W (2015). The effect of compressive loading magnitude on in situ chondrocyte calcium signaling. Biomech Model Mechanobiol.

[53] Trindade MC, Shida J, Ikenoue T, Lee MS, Lin EY, Yaszay B (2004). Intermittent hydrostatic pressure inhibits matrix metalloproteinase and pro-inflammatory mediator release from human osteoarthritic chondrocytes in vitro. Osteoarthr Cartil.

[54] Mawatari T, Lindsey DP, Harris AH, Goodman SB, Maloney WJ, Smith RL (2010). Effects of tensile strain and fluid flow on osteoarthritic human chondrocyte metabolism in vitro. J Orthop Res.

[55] Erickson GR, Northrup DL, Guilak F (2003). Hypo-osmotic stress induces calcium-dependent actin reorganization in articular chondrocytes. Osteoarthr Cartil.

[56] Chen J, Yuan Z, Liu Y, Zheng R, Dai Y, Tao R (2017). Improvement of in vitro three-dimensional cartilage regeneration by a novel hydrostatic pressure bioreactor. Stem Cells Transl Med.

[57] Degala S, Williams R, Zipfel W, Bonassar LJ (2012). Calcium signaling in response to fluid flow by chondrocytes in 3D alginate culture. J Orthop Res.

[58] Westacott CI, Whicher JT, Barnes IC, Thompson D, Swan AJ, Dieppe PA (1990). Synovial fluid concentration of five different cytokines in rheumatic diseases. Ann Rheum Dis.

[59] Denoble AE, Huffman KM, Stabler TV, Kelly SJ, Hershfield MS, McDaniel GE (2011). Uric acid is a danger signal of increasing risk for osteoarthritis through inflammasome activation. Proc Natl Acad Sci U S A.

[60] Bertazzolo N, Punzi L, Stefani MP, Cesaro G, Pianon M, Finco B (1994). Interrelationships between interleukin (IL)-1, IL-6 and IL-8 in synovial fluid of various arthropathies. Agents Actions.

[61] Kagari T, Doi H, Shimozato T (2002). The importance of IL-1β and TNF-α , and the noninvolvement of IL-6, in the development of monoclonal antibody-induced arthritis. J Immunol.

[62] Na Y, Bai R, Zhao Z, Wei Y, Li D, Wang Y (2017). IL1R1 gene polymorphisms are associated with knee osteoarthritis risk in the Chinese Han population. Oncotarget.

