# Supplementary material for: Compressive mechanical stress enhances susceptibility to interleukin-1 by increasing interleukin-1 receptor expression in 3D-cultured ATDC5 cells
Source: BMC Musculoskelet Disord. 2021 Mar 1;22:238. doi: 10.1186/s12891-021-04095-x (PMC7923672; doi:10.1186/s12891-021-04095-x)
Supplement: Supplementary file 1 — Additional file 1. [file 12891_2021_4095_MOESM1_ESM.pdf]

Supplementary Figure

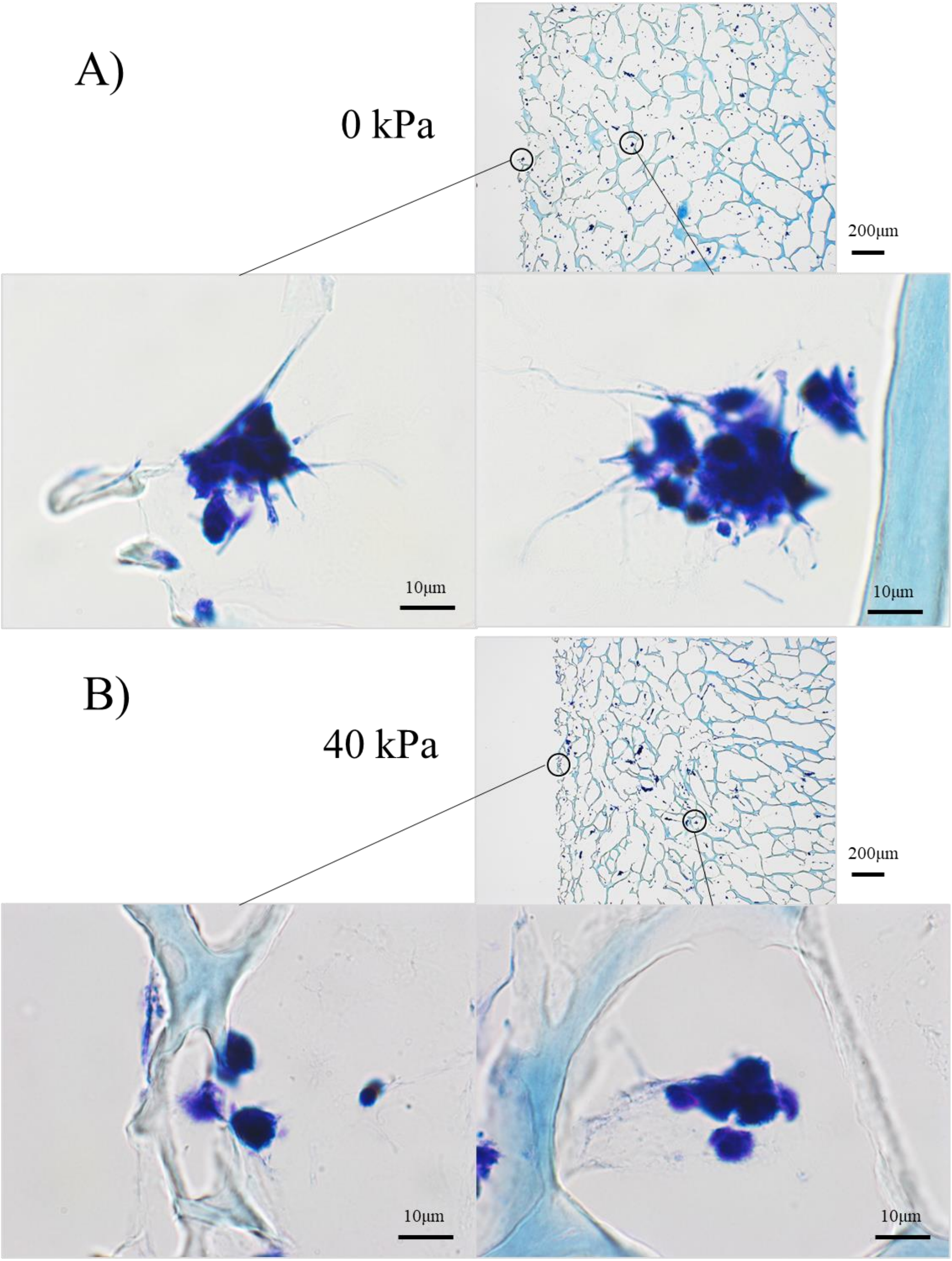

At 0kPa, cells extended their cell protrusions in 3D-cultured constructs (A). At 40 kPa, the cell protrusions disappeared and many cells slightly exhibited a round shape (B).
